# Sleep disorders among prison officers in Poland

**DOI:** 10.1192/j.eurpsy.2022.2101

**Published:** 2022-09-01

**Authors:** A. Piotrowski, E. Ewa Sygit-Kowalkowska

**Affiliations:** 1 University of Gdansk, Institute Of Psychology, Gdańsk, Poland; 2 Kazimierz Wielki University in Bydgoszcz, Faculty Of Psychology, Bydgoszcz, Poland

**Keywords:** job burnout, prison officers, Insomnia, coping with stress

## Abstract

**Introduction:**

Workplace conditions have a documented effect on employee health including sleep. Occupational stress and burnout are more frequent among penitentiary personnel than the general population.

**Objectives:**

The aim of the current study was to examine the phenomenon of insomnia and its relationship with occupational burnout in a sample of Polish prison officers.

**Methods:**

The study was carried out on a sample of Polish prison officers using the Athens Insomnia Scale (AIS), the Coping Orientation to Problems Experienced (COPE), and the Oldenburg Burnout Inventory (OLBI).

**Results:**

showed that the Polish prison officers exhibited early symptoms of insomnia. Sleep disorders had a significant role in developing occupational burnout. Coping strategies such as help-seeking and engagement were revealed to have a mediating role in the relationship between insomnia and occupational burnout dimensions. The coping strategy of help-seeking was the only predictor of insomnia.

**Conclusions:**

The results can constitute a significant argument for health promotion campaigns highlighting sleep hygiene directed at penitentiary personnel. A research model created for the purposes of future studies would allow for measuring the frequency of health behaviors, including the general category of preventive behaviors. The study warrants continuation.

**Disclosure:**

No significant relationships.

